# The Progression in Developing Genomic Resources for Crop Improvement

**DOI:** 10.3390/life13081668

**Published:** 2023-07-31

**Authors:** Pradeep Ruperao, Parimalan Rangan, Trushar Shah, Vivek Thakur, Sanjay Kalia, Sean Mayes, Abhishek Rathore

**Affiliations:** 1 Center of Excellence in Genomics and Systems Biology, International Crops Research Institute for the Semi-Arid Tropics (ICRISAT), Hyderabad 502324, India; 2 ICAR-National Bureau of Plant Genetic Resources, PUSA Campus, New Delhi 110012, India; r.parimalan@icar.gov.in; 3 International Institute of Tropical Agriculture (IITA), Nairobi 30709-00100, Kenya; tm.shah@cgiar.org; 4 Department of Systems & Computational Biology, School of Life Sciences, University of Hyderabad, Hyderabad 500046, India; vivek22@uohyd.ac.in; 5 Department of Biotechnology, Ministry of Science and Technology, Government of India, New Delhi 110003, India; sanjay.kalia@nic.in; 6 Excellence in Breeding, International Maize and Wheat Improvement Center (CIMMYT), Hyderabad 502324, India

**Keywords:** sequencing technologies, plant genomes, pan-genomes, assemblies, bioinformatics tools, databases, big data, artificial intelligence, machine learning

## Abstract

Sequencing technologies have rapidly evolved over the past two decades, and new technologies are being continually developed and commercialized. The emerging sequencing technologies target generating more data with fewer inputs and at lower costs. This has also translated to an increase in the number and type of corresponding applications in genomics besides enhanced computational capacities (both hardware and software). Alongside the evolving DNA sequencing landscape, bioinformatics research teams have also evolved to accommodate the increasingly demanding techniques used to combine and interpret data, leading to many researchers moving from the lab to the computer. The rich history of DNA sequencing has paved the way for new insights and the development of new analysis methods. Understanding and learning from past technologies can help with the progress of future applications. This review focuses on the evolution of sequencing technologies, their significant enabling role in generating plant genome assemblies and downstream applications, and the parallel development of bioinformatics tools and skills, filling the gap in data analysis techniques.

## 1. Introduction

With more than 40 years of remarkable DNA sequencing improvements, today, the development of cost-reducing and higher throughput sequencing technologies, along with relevant bioinformatics tools, have made it possible to produce high-quality genome assemblies in a much-reduced timeline, which has subsequently led to the mapping of the genetic variations in thousands of individuals, providing genetic insights into population histories and domestication events. The multinational and multi-institutional consortium the Earth BioGenome Project (EBP) aims to unify the phylogenetic networks across all eukaryotic life derived from their complete de novo genomes [1,2]. This illustrates how far the advancement and standardization of genome data generation, assemblies, storage, retrieval, and analysis have developed, with more expected and required with the generation of massive genomic data from species bridging the phylogenetic gaps between currently sequenced genomes.

Complete reference genome assemblies of the entire plant kingdom will open new scientific views on the evolution and speciation events on earth and genetic control of plant traits, both at intra- and inter-species levels. They will also enhance the understanding of how plants function in ecosystems, lead to the discovery of natural botanical compounds for human medicine, and will aid an increase in food production to curb global hunger while respecting planetary boundaries and adapting to climate change.

Here, we provide an overview of the improvements in sequencing technologies, the development of the associated bioinformatics tools, and advancements in plant genomics. We also outline the progress achieved in assembling plant genomes, sequence technologies, and assemblers used to contribute towards crop improvement.

## 2. Genome Sequencing Milestones

Over 40 years of consistent development of reliable sequencing technology emerging to make considerable progress in accuracy, cost, and reduced sequencing time has been improved.. From first-generation to third-generation sequencing, the combined technologies developed have significantly increased the read length, improved quality, and provided massive increases in throughput with significant cost reductions. However, currently, second-generation (also called next-generation) sequencing technology dominates. 

### 2.1. First-Generation Sequencing (FGS)

It is during this phase that the sequencing process advanced as a technology to help understand the genetic basis behind the phenotype. This first-generation sequencing technology is based on the dideoxynucleotide chain termination method developed by Sanger and Coulson in 1975, commonly known as the Sanger method [3] and nucleobase-specific partial chemical modification of DNA in Maxam–Gilbert sequencing [4]. The first genome sequence for Phage X174 was generated in 1977 using a variant of this method. The automated Sanger method (through capillary electrophoresis in 1980) was an essential improvement and aided the completion of the Human Genome Project in 2001. The merit of this technology was that it produced a read length of around 1 kb with 99.999% accuracy, but the drawback was its high cost, short run length, and low throughput. 

### 2.2. Second-Generation Sequencing (SGS)/Next-Generation Sequencing (NGS)

While the Sanger method was continuously popular, particularly for accurate sequencing of specific sequences, such as genes, many other sequencing technologies emerged at around the same time, such as (i) the pyrophosphate sequencing used by Roche for the 454 sequencing platform (the first major successful commercial SGS technology), (ii) the ligation enzyme method used for the SOLID technique by ABI sequencing company, (iii) single-molecule sequencing with HeliScope from Helicos Biosciences, and (iv) DNA colony sequencing technology from Illumina.

**Figure 1:**
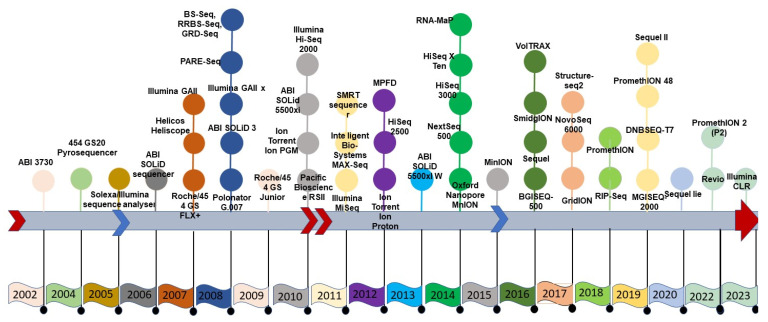
Timeline of the advancement of sequencing technology.

SGS sequencing was conducted in either a stepwise iterative process or in a real-time manner, producing a combination of qualitative and quantitative sequence information, which was not possible with FGS data. The second generation of sequencing technology was symbolized by several approaches, all fundamentally based on parallel data production with individual sequences identified by position on a flow cell or microarray. Roche’s 454, Illumina’s Solexa, Hiseq technology, and ABI’s Solid technologies not only reduced sequencing costs but also increased the speed of sequencing [5]. The thirteen-year duration of the human genome project using Sanger sequencing would now take just one week with SGS technologies to generate the raw sequence data—although assembly remained a significant computational problem. Sequencing throughput has increased with SGS technologies, but the read length is often much shorter than in the first generation. The specific technologies of SGS include the Ion Torrent technology that directly produces digital nucleotide sequence information on a semiconductor chip [6]. It is possible to generate such sequence information with several versions of the Ion Torrent platform, such as the Ion Personal Genome Machine (PGM) System, Ion Proton System and Ion S5 system, and ION S5 XL systems. The Roche/454 Life Sciences introduced several sequencers in the form of GS, GS 20 run, and GS FLX titanium. Similarly, Illumina sequencing supports a variety of protocols with varying levels of throughput, including MiniSeq [7], MiSeq [8], NextSeq (https://doi.org/10.48550/arXiv.1711.11004), HiSeq [9], and NovaSeq models (Figure 1) (Table 1). 

### 2.3. Third-Generation Sequencing

Third-generation sequencing (TGS) technologies have focused on increasing the read length while maintaining the sequencing throughput. The single-molecule real-time sequencing-by-synthesis (SMRT) technology from Pacific Biosciences introduced read lengths of single DNA fragments exceeding 10 Kb, and long sequences are particularly useful for *de novo* genome assemblies, especially where genomes are large or contain repetitive DNA, as is the case with many plants [10]. Pacific Biosciences (PacBio) has commercialized two sequencing systems, RSII model and Sequel II, producing high-fidelity (HiFi) reads with more than 99% accuracy [11], and Revio is an advanced platform to generate HiFi reads at a higher throughput (15X. SMRT now enables the generation of very long reads of lengths over 30 Kb to 50 Kb. 

With continual upgrades in sequencing chemistry and technology, it is possible to generate longer reads of over 100 Kb in length. Nanopore is a technology that takes a different approach to sequencing via synthesis adopted by PacBio. This technology (proposed in 1990 and commercialized by Oxford Nanopore Technologies, ONT) decodes the DNA molecule by detecting electrical fluctuations as a nucleic acid molecule passes through a small diameter biological “pore”. Continuous sequences from single molecules up to 500 Kb have been reported, although generally, a single molecule sequence is likely to average around 20 Kb [12]. By having multiple parallel pores and very rapid processing, it is possible to obtain hundreds of gigabases of nucleotide sequences at a low cost. Early iterations of the technology had relatively poor accuracy, and while the accuracy has improved (partly through the development of software specifically for interpreting nanopore signals), a nanopore is often corrected in practice by using highly accurate short Illumina reads before being used for genome assembly. Nanopore technology comes in different versions, including MinION, benchtop GridION, VolTRAX, and high-throughput PromethION [13]. Next, 10× Genomics is another long-read sequence technology (www.10xgenomics.com) integrated with GemCode technology supplied with the Supernova2 genome assembler. This technology was specifically designed for diploid and low-complex genomes, such as *Corylus avellana*, where its implementation produced a chromosome-level genome assembly [14].

Similarly, optical mapping and Dovetail Hi-C technologies are useful to complete the ordering of various DNA contigs in a genome by creating a visual physical map along large DNA molecules, which assist in correlating a DNA sequence with a physical location [15,16]. This technology was further improved by using nanofluidic methods, and image capture and processing have further improved optical mapping [17,18]. The Bionano technology was commercially developed and made available to process samples through Bionano Genomics (San Diego, CA, USA) (https://bionanogenomics.com/products/) and OpGen (http://www.opgen.com/about-us/opgen-overview/).

**Table 1:** Sequencing technologies and applications in the SGS era.

Sequencer/Technology	Applications	Reference
ChIP-Seq	Protein-DNA interactions (using chromatin immunoprecipitation)	[19]
DNA-Seq	A genome-derived sequence	[20]
RIP-Seq, CLIP-Seq, HITS-CLIP	Protein–RNA interactions	[21]
RNA-Seq	RNA (that is, the transcriptome)	[22]
RAD-seq	Restriction site-associated DNA sequencing	[23]
TRAP	Genetically targeted purification of polysomal mRNAs	[24]
Global run-on sequencing (GRO-Seq)	Transcript analysis	[25]
Reduced representation bisulphite sequencing (RRBS-Seq)	Genome methylation	[26]
Bisulfite sequencing (BS-Seq)	Genome methylation	[27]
Parallel analysis of RNA ends sequencing (PARE-Seq)	microRNA target discovery	[28]
Targeted DNA-Seq	A subset of a genome (for example, an exome)	[29]
Methyl-Seq	Sites of DNA methylation, genome-wide	[30]
Targeted methyl-Seq	DNA methylation in a subset of the genome	[31]
Hi-C	Three-dimensional genome structure	[32]
Chia-PET	Long-range interactions mediated by a protein	[33]
Ribo-Seq	Ribosome-protected mRNA fragments (that is, active translation)	[34]
Synthetic saturation mutagenesis	Functional consequences of genetic variation	[35]
MAINE-Seq	Histone-bound DNA (nucleosome positioning)	[36]
FRT-Seq	Amplification-free, strand-specific transcriptome sequencing	[37]
PARS	Parallel analysis of RNA structure	[38]
Deep protein mutagenesis	Protein binding activity of synthetic peptide libraries or variants	[39]
Repli-Seq	Replication	[40]
DNase-Seq, Sono-Seq, and FAIRE-Seq	Active regulatory chromatin (that is, nucleosome-depleted)	[41]
NET-Seq	Nascent transcription	[42]
Immuno-Seq	The B-cell and T-cell repertoires	[43]
PhIT-Seq	Relative fitness of cells containing disruptive insertions in diverse genes	[44]
Nacent-Seq	Transcription	[45]
ChIRP-Seq	Genome localization	[46]
Massively parallel functional dissection sequencing (MPFD)	Enhancer assay	[47]
Assay for transposase-accessible chromatin using sequencing (ATAC-Seq)	Open chromatin	[48]
Structure-Seq	RNA structure	[49]
RNA on a massively parallel array (RNA-MaP)	RNA–protein interactions	[50]
SEQ-500	Genome sequencer	[51]
RNA immunoprecipitation sequencing (RIP-Seq)	RNA–protein interactions	[52]
HiSeq 2000/2500/4000/X10	Genome sequencer	www.illumina.com
MGISEQ-2000	Genome sequencer	www.en.mgi-tech.com
NovaSeq 6000	Genome sequencer	www.illumina.com
PacBio Sequel/II/HiFi	Genome sequencer	www.pacb.com
Nanopore PromethION/MinION	Genome sequencer	www.nanoporetech.com
MiSeq	Genome sequencer	www.illumina.com
TruSeq	Genome sequencer	www.illumina.com
DNBSEQ-T7	Genome sequencer	www.en.mgi-tech.com
MeDip-Seq/DIP-Seq	Methylated DNA immunoprecipitation sequencing	www.illumina.com

## 3. Plant Genomic Resources (Big Data Generation)

Sequencing technologies, mainly using high-throughput NGS sequencers, generate significant amounts of data. For example, the recent sequencer from Illumina (NovaSeq 6000) has a higher output than the earlier generation of sequencing machines producing between 1300–20,000 million reads (65 Gb to 3 Tb). The long reads from PacBio reach up to a maximum of 300 Kb, and the data generated with Sequel I, II (CLR), II (HiFi) range from 0.5 million to 400 million reads (15 Gb to 100 Gb), with the nanopore sequencing technology (Minion and Promethion) sequencing ranging from 2.5–12 million reads (40 Gb to 180 Gb). 

With this capacity, sequencing land plants having a wide range of genome size DNA content can, in theory, possibly generate good coverage of the entire genome sequence data. For example, the corkscrew plant *Genlisea margaretae* with a 1C value of 0.07 pg (65 Mb) and the canopy plant *Paris japonica* with a 1C value of 152.2 pg (148.9 Gb) are equally accessible in terms of raw sequence generation and coverage [53] (https://cvalues.science.kew.org/). Generating several-fold coverage of genomic data produces potentially massive datasets, ranging from Gb to Tb of sequence information. Depending on the scope of the project, handling such large datasets is a major concern for small (or even big) research labs. Decades ago, geneticists were mostly involved in lab work; now, the most limiting factor is the analysis of the data to derive meaning or interpretation out of it using computational tools. Understanding the algorithms and processing the data are a crucial part of genetics and genomics data analysis when searching for biological meaning. 

Genomic sequencing is a field where handling big data and its processing requires a suitable storage and data transfer platform, such as is present in cloud technologies. These are extensively applied to enhance the availability of the data to all researchers in a project and indeed researchers worldwide. The genome sequence data generated for a crop genome project are immense; for example, a single Sorghum genome sequence contains over 50 gigabytes of raw data (depending on the data format generated), and processing the data for large population-wide studies, such as finding deeper scientific insights, marker–trait association, analyzing diversity, domestication, and assessing data from gene-editing technologies, requires robust storage and computing capacities. 

To maintain the uniformity of the data in the global databases, the members’ databases (GenBank, EMBL, DDBJ, CNGBdb, IBDC) of the International Nucleotide Sequence Database Collaboration (INSDC) [54] share and update genomic data periodically.

**Figure 2:**
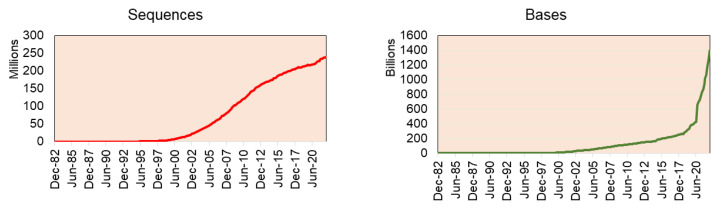
The sequences deposited in Genbank by year indicate that genome sequence projects have increased steadily since 2002, while absolute data have exploded since 2020.

The recent stats release of GenBank reports having 16.7 trillion nucleotide bases for 1.7 million whole genome sequences (as of June 2022) (GenBank and WGS Statistics (ncbi.nlm.nih.gov)) (Figure 2). Of which, green plant data (Viridiplantae) alone have 93.8 million sequences from 2324 genomes (including variants of the same plant species genome), including genomic DNA/RNA for 33.4 million sequences, mRNA for 41.5 million sequences, and rRNA for 80,709 sequences. 

With the increasing complexity of genomic data themselves, the major databases also integrate other genomic features and provide tools to search and retrieve these datasets. The Entrez system of NCBI is one such tool allowing users to search, view, and download the sequences from GenBank. Other modes of data accessibility allow for downloading from the FTP site (ftp.ncbi.nlm.nih.gov) or downloading data programmatically with the provided public API to the Entrez system (https://eutils.ncbi.nlm.nih.gov). 

Numerous databases have been developed for genomic data to suit a variety of different purposes (Table 2). Based on the data catchment of the database, the database is as big as a global repository holding the sequences of all species, like Ensembl Plants, the National Centre for Biotechnology Information (NCBI), PlantGDB, the Plant Genome Database Japan (PGDBj), to medium size databases hosting only plant genome assemblies/annotations, like Phytozome and the Legume Information System (LIS) (https://www.legumeinfo.org), to smaller databases containing crop/plant-specific information, such as for the chickpea SSR database (https://cegresources.icrisat.org/CicArMiSatDB/index.html) [55] and chickpea SNP and indel database (https://cegresources.icrisat.org/cicarvardb/) [56]. However, the medium to smaller databases are limited to the scope of species-level data, like the LIS and proposed angiosperms database [57], and may do not need to use powerful bioinformatics tools and computational resources to explore the terabytes of genomic data, and many such databases were earlier discussed in [58].

## 4. Plant Genome Assemblies

Genome assembly refers to aligning the small fragments of a DNA sequence to reconstruct the genome sequence in the original order and orientation. High-throughput sequencing through first- and second-generation sequences has enabled the assembly of many plant genomes. The highly fragmented genome assemblies generated with short reads have been improved with long read sequence assemblies, simplifying and improving the ability to generate chromosome-level assemblies with reduced reliance on dedicated research experts.

Thanks to the NGS technology and increased computational power, the standard of the genome assemblies available has improved significantly. Genomics has accelerated its growth in the past decade from draft-level genome assemblies to reference-level genome assemblies [78,79,80]. 

**Figure 3:**
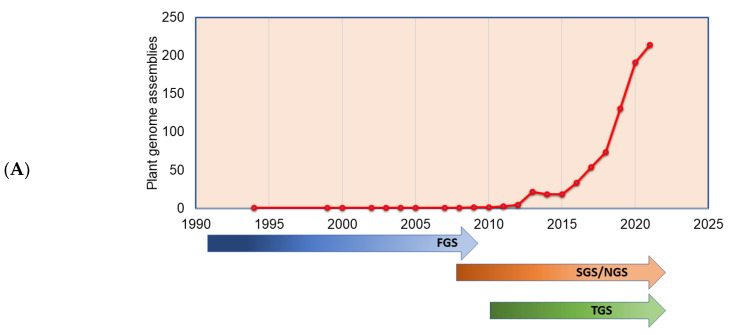

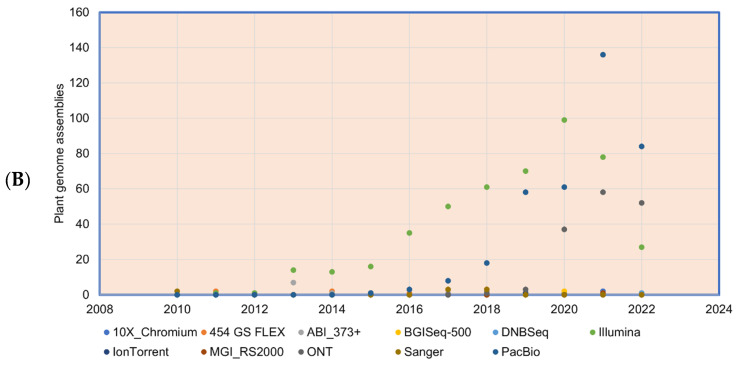
Plant genomes assembled in (**A**) different generations of sequencing technology and (**B**) sequence technologies used for plant genome projects (the genome assembly projects mostly use multiple sequencing techniques to gain higher accuracy, and the data point indicates the count of the number of times the sequencing technique has been reported in a particular year for genome assembly). The plant genome assemblies stats are derived from [119].

The plant genomes assembled in the FGS era faced significant throughput issues and were limited by a read length of around 1 Kb. This necessitated approaches such as BAC-end reads and BAC barcoding to allow contigs to be linked and positioned throughout the genetic mapping. The plant genomes assembled in the FGS era are far fewer than the genomes assembled in the SGS and TGS sequencing technology era (Figure 3A), primarily due to the lower throughput and high cost of FGS. The situation changed sharply with SGS, as the volume of the sequence (although not the length) was significantly increased. Long-read sequence technologies play a crucial role in genome assembly projects, which helps in scaffolding the contig sequences, and thus many genome projects were initiated with combined SGS and TGS technologies (Figure 3B). With the advent of advanced sequence technologies such as PacBio HiFi sequencing, which produces a 10 to 30 Kb circular consensus sequence, thus reducing error rates (CCS) [11], Oxford Nanopore long-read protocols [81], Hi-C scaffolding [32], and optical mapping technologies, such as Bionano [82], it is possible to assemble complex genomes. The emerging third-generation sequence data have boosted the genome assembly quality to build a chromosome-level assembly by overcoming the limitation of short reads assembly, particularly in plants, where islands of repeat sequences need to be bridged between the gene-rich regions of the chromosomes. With the low-cost and high-throughput sequence data generations, at least 1143 plant reference assemblies have been published (www.plabipd.de) (Supplementary Table S1). Based on the availability of funds and the feasibility of applying high-volume sequence data generation, multiple individuals of the same species were *de novo* assembled, e.g., potato [83], or the genome assembly of the same varieties improved, such as for chickpea [84,85] and sesame [86]. The development of long-read technologies as part of the TGS allowed for a relatively simple assembly of smaller genomes. With optical and chromatin-based methods, such as Bionano and HiC, far more comprehensive and larger genome assemblies are now possible, which are based on a range of techniques, including the integration of scaffolds into the chromosome through genetic mapping.

In recent years, gold-standard and platinum-standard chromosome-level genome assemblies are being achieved in prominent model crop plants [87,88,89,90,91,92]. Here, gold-standard assembly refers to cases where the number of superscaffolds matches the number of haploid chromosomes, yielding a chromosome-level assembly; a platinum-standard assembly refers to a telomere-to-telomere (T2T) assembly with the final scaffolds matching the number of haploid chromosomes. This era has led to gold- or platinum-standard assemblies in crop plants, and publications meeting these standards are continuing to appear [93]. The importance of having platinum-standard reference genome assemblies and the importance to compare cultivated species with wild relatives of rice is documented [94].

Chromosome-level genome assemblies were initiated with Arabidopsis in 2000 [95] and later with rice in 2005 [96]. These assemblies were generated with the traditional, expensive, and low-throughput Sanger sequencing method. With current third-generation sequencing (such as PacBio, HiFi, Hi-C, and optical mapping methods), it is possible to generate chromosome-level pseudomolecules [97]. With PacBio sequence data, a chromosome-level assembly was first achieved for Arabidopsis [98] followed by Oropetium [99]. Similar to the PacBio long reads, ONT generates around 200 Kb length reads highly suitable for bacterial genomes assembly [100]. Synthetic long reads (SLR) are long reads generated from Illumina short-read data to assemble long reads [101]. In total, 113 plant species have the chromosome-level genome assemblies published (as of the end of 2022) (www.plabipd.de) of the total assembly number of 1143 flowering plants, and 125 are non-flowering plants (Supplementary Table S1). Most of these near-complete plant genomes were produced with sequence data generated from multiple technologies. The long-read 10× Genomics with short-read Illumina data were used to assemble the blueberry genome [102]. PacBio and Hi-C sequence technology were used for assembling the octoploid sugarcane genome [103], allotetraploid peanut [104], and teff [105]. 

Several novel technologies have emerged (such as optical mapping [106]), the Irys system by BioNano Genomics (www.bionanogenomics.com) and chromosome conformation capture sequencing (Hi-C) [32]) to improve the scaffolding without depending on genetic mapping. However, these advances in genome assembly have recently improved further to generate the telomere-to-telomere (T2T) assemblies, as first implemented in 2020 for the X chromosome sequence of the human genome [107] and later adapted to plants, such as Arabidopsis [108,109], rice [110], and banana [111] (Table 1). The combined integration of PacBio and modified Hi-C protocol as Dovetail Genomics has improved the assembly contiguity for *A. alpina* [112]. The high-resolution gap-free T2T genome assemblies ensure the capture of all the repetitive sequences and genomic variants without any misassemblies.

The greatest bioinformatics challenge for sequencing plant genomes was repetitive sequences, leading to sequencing errors and unrecognizable assembling errors at earlier stages of assembly computation. As the plant genome size and ploidy or repeat content increases, the complexity of assembly of the sequence reads correctly also increases, and thus the assembly programs used in these genome projects needed increasingly sophisticated strategies (such as chromosome flow sorting methods used in wheat) to handle such challenges. Additionally, handling the terabytes of sequence data and storage and managing the computing clusters and complexity of the algorithms also need to be addressed.

In addition to improving the quality of reference genomes to platinum-standard, present-day technologies paved the way for the transformational shift from the representative single genotype’s genome sequence to the pan-genome sequence as a reference for a better understanding of the variability present within a species [113]. The advantages of the pan-genome reference are being realized in generating novel insights and the identification of the genes or genomic regions underlying the important agronomical traits and domestication process [86,114,115,116,117,118].

## 5. Genome Assemblers

**Figure 4:**
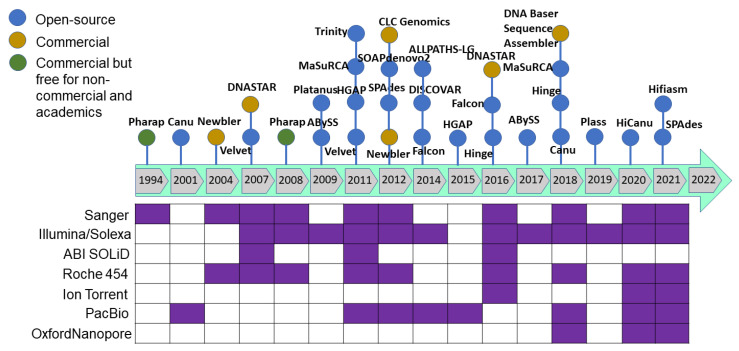
The most commonly used genome assemblers and their release timeline to analyze the sequence generated from variant sequencing technologies.

**Figure 5:**
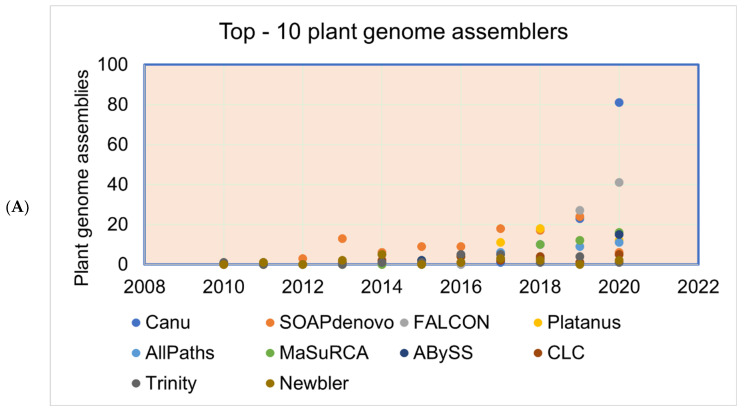

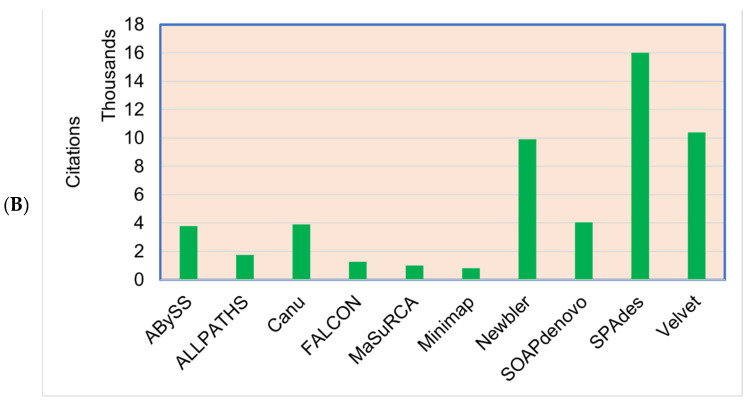
(**A**) The most commonly used genome assemblers for the plant genome assemblies’ projects and the (**B**) citations of the assemblers.

As sequencing technology evolved, assembly approaches also had to evolve. The Celera Assembler and Arachne assemblers were developed to handle genomes of the fruit fly (*Drosophila melanogaster*) and human genome in 2000–2003; later, AMOS was launched under an open-source framework. These assemblers were developed based on overlap–layout–consensus on an overlap graph [120] in which the nodes were the reads and the edges represented the shared sequence between reads. This type of assembler is suitable for assembling FGS technology sequencing reads produced by the dideoxy termination method (Sanger sequencing). As massively parallel high-throughput sequencing technology was developed to produce millions of bases (in SGS), the read size became smaller and more error-prone with higher genome coverage. The leading Illumina technology of SGS/NGS sequencing technology yields 35–150 bp length paired-end reads from fragments with a 200–300 bp insert size. Such high-throughput data required a new approach, and thus *de Bruijn* graph-based assembly was developed [121,122] where the nodes represent fixed-length strings drawn from a larger set of strings, and the edges represent perfect shared sequences. However, *de Bruijn* graph-based assemblers have difficulties handling sequencing errors and need high computational power (100+ Gb of memory). The challenge with uneven genome coverage and reads too short to span repeated regions can be addressed by a combination of many short reads and fewer longer reads or mate–pair reads (Sanger, 454 and Illumina sequencing methods). Multiplex *de Bruijn* graphs automate the assemblies of long HiFi reads [123], and the recently updated Minimap2 version can be used for long read assembly [124]. Newbler was the first assembler released in 2004 to assemble the 454 sequence data followed by a hybrid version of the MIRA assembler for 454 and mixed with Sanger reads. After upgrading the Illumina sequence technology to produce from the initial 36 base-length read to reads over 100 bases in length, the produced sequence was suitable for *de novo* assembly. After the release of the SHARCGS assembler for Solexa reads, other assemblers were released and became the most popular assembly tools (Figure 4 and Figure 5).

Plant genome assembly was initiated with *Arabidopsis thaliana* in December 2000 [95] where the approach relied on overlapping bacterial artificial chromosome (BAC) clones which were end sequenced and the same approach was applied to the crop plant rice [125,126]. Later, the emerging whole genome shotgun (WGS) strategy was applied to black cottonwood [127]. This was where more difficulties and challenges were faced to assemble the short sequence reads, which resulted in a more fragmented assembled genome sequence followed by two versions of the grapevine genome sequence in 2007 [128,129]. A hybrid approach was adopted to sequence the cucumber with Illumina and Sanger sequencing technology, indicating the feasibility of using this approach for plant genome sequencing [130]. With the change in technology, 454 combined with the Sanger sequencing approach was applied to the genomes of apple [131], cocoa [132], and muskmelon [133]. In 2011, the first plant genome was sequenced using SGS technology combining 454, Illumina, and the SOLID platform for strawberry [134], Chinese cabbage [135], potato [136], chickpea [137], pigeonpea [138], and watermelon [139].

The advances in sequencing technology (SGS and TGS) and assembly approaches have removed the limitation of genome sequencing for not only the crops with small genome sizes but also enabled sequencing and assembly of large genome crops, like wheat (~17 Gbp) [87,140,141], barley (5.1 Gbp) [142], rye (~7–8 Gbp) [143], and tea (~3.8–4.0 Gbp) [144], which are important for animal feed and human nutrition. 

The genome assembly quality has improved as the sequencing technologies and assembling tools improved (Figure 3, Figure 4 and Figure 5), especially when combined with the utilization of multiple sequencing technologies of TGS, for example.

The initial assembly version of the sorghum genome assembly released in 2009 [145] with shotgun sequencing and BAC libraries data captured 738.5 Mb of sequences in 12,873 contig sequences (scaffolded to 3304 sequences), which is more fragmented compared to the chromosome-scale assembly of the sorghum genome using nanopore sequencing and optical mapping data that produced a hybrid assembly made of 29 scaffolds capturing the 661.16 Mbps [146].

For a large genome (~8 Gb) rye (Secale cereale), initially, a virtual linear gene order model (22,426 genes) was established with high-throughput transcript mapping and chromosome survey sequencing [147]; following reference genome assembly with a shotgun, *de novo* genome assembly produced 1.29 million scaffolds, capturing 2.8 Gbp of sequence [148] and later chromosome-scale genome assembly with 10×, HiC, Bionano optical genome mapping, and chromosome-specific shotgun (CSS) reads produced 6.74 Gb (of estimated 7.9 Gb) [149].

In addition to the chromosome-scale assemblies, TGS has enabled the assembly of polyploid genomes, such as bread wheat [87], potato [150], and peanut [151].

## 6. Advancements in Plant Genomics

With the emerging sequence technology and bioinformatics tools, it is possible to assemble a nearly complete genome sequence. With cytogenetic advances to measure the genome size (such as flow cytometry), a genome size estimation is a useful first step in a complete genome sequencing project. The amount of sequencing data required to produce a given level of coverage depends on the 1C amount of DNA per cell (including ploidy level), and for most species, this can be found in the Kew Plant Genome Database. Most plant genome assemblies are smaller than the cytogenetic genome estimation size; this may be because of assembly errors or difficult-to-approach genomic regions, like centromeric and repetitive regions in the plant genome, where assemblers struggle (physical maps, such as Bionano, resolve such issues). Some of the assembled plant genome sizes are quite close to the cytogenetic estimated size, indicating the assembler has captured the majority of the genome content. Assemblies above the estimated size, however, may need refinement to reduce contaminants or alter the assembly parameters. 

**Figure 6:**
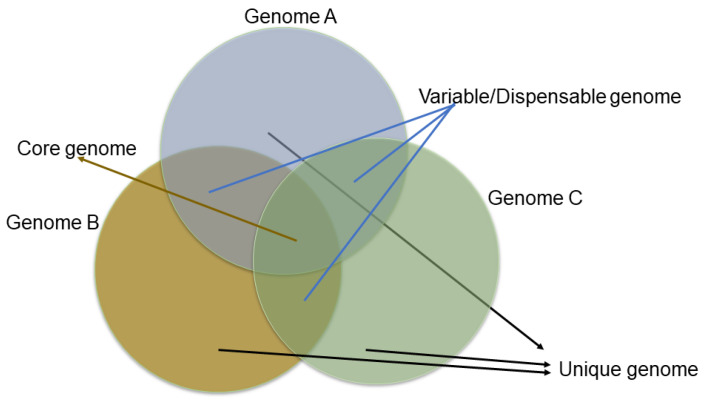
Venn diagram showing core and accessory genes.

The genome assembly provides the coordinate system for the gene models and other genomic features, like SNPs, Indels, SSRs, etc. Predicting the gene models with ab initio gene findings and supporting evidence in the form of RNA data increases the accuracy. However, this may not list out the complete complement of genes of the species for which resequencing a wide range of diverse accessions will reveal more genes that are genotype-specific. For example, the resequencing of >1000 wild and cultivated rice accessions has predicted the presence of thousands of genes with lower sequence diversity in cultivated rice, indicating a rice domestication genetic bottleneck [114,152]. Moreover, genetic diversity is often reduced during domestication, and resequencing a single individual may not capture the species-wide gene content. Thus, the concept of the pan-genome was developed and adapted to plants’ genomes to identify the species-wide gene content. The core genome is usually defined as the housekeeping genes (which must be present for the organism to survive and reproduce) and the variable/dispensable genes (these genes are present or absent in a particular cultivar/accession of a species) that exhibit the gene diversity or variability in a species (Figure 6). Thus, the first plant pan-genomes appeared in 2007, describing the variable genes in rice and maize genomes, and were later adapted to a wide range of plant genomes [153], including banana [154], white lupin [155], barley [156], wheat [156], wheat panache [157], and sorghum [158] (Table 3).

**Figure 7:**
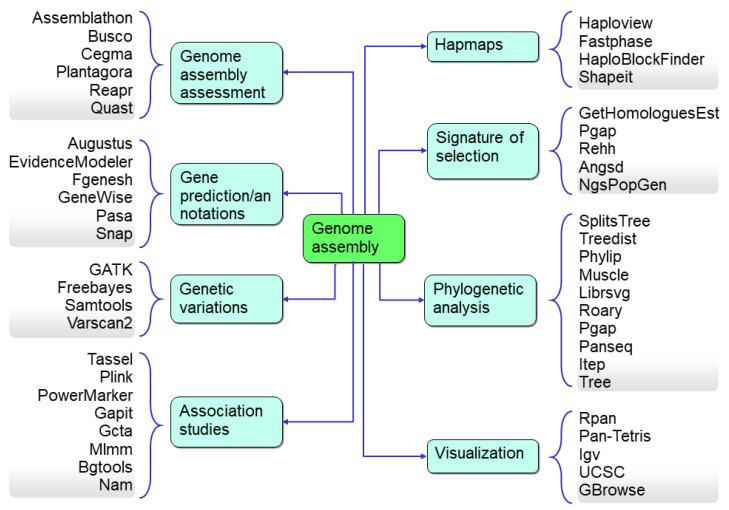
The downstream analysis and the associated bioinformatics tools used for the data analysis.

The most commonly used downstream analysis with pan-genome assemblies is to identify the genetic variation of any DNA segment in a genome or a gene (including gene fragments) that can be used as a marker for genotyping. Bioinformatics resources enhancing crop genomics for downstream analysis include copy number variations (CNV), identification of variations based on the length (SNP, SSR, Indels), a set of SNPs used as a unit in the form of a haplotype to increase the resolution of GWAS, k-mer analysis, linkage disequilibrium (LD), presence–absence variations, pan-genome-wide association studies (PWAS), genotyping-by-sequencing, reduced representation sequencing, domestication, and diversity analysis (Figure 7). With these bioinformatics tools, the genomic data also assists plant phylogenomic research with useful information, such as genome diversity and speciation events. Therefore, bioinformatics has become a most essential part of plant genomics research.

High-throughput genotyping enables the genotyping of thousands of targeted loci (genetic markers) on thousands of samples. Depending on the number of markers and the sample size, different genotyping techniques can call genotypes in different ranges. Some of the technologies include Illumina golden gate, Affymetrix SNP, reduced-representation genome sequencing, exome-seq, Fluidigm (https://investors.fluidigm.com/node/13686/pdf), IntelliQube (https://www.myebpl.com/intelliqube.html), MassARRAY [185], MassEXTEND, GeneChip [186], APEX-Seq [187], BeadARRAY (https://www.illumina.com/science/technology/microarray.html), TaqMan [188], and DArT (https://www.diversityarrays.com/). Genotyping by sequencing (GBS) is a highly multiplexed system for constructing reduced representation libraries from the sequencing platform with low-cost, reduced sample handling with no need for a reference genome. GBS (including the single digest RAD and double digest RAD and skim-sequencing) are tools for genomics-assisted breeding in a range of plant species through the applications of SNPs identification, gene/QTL mapping, molecular diversity, GWAS, construction of high-density genome maps, haplotype maps, phylogenetics, identification of candidate genes, genetic linkage analysis, molecular marker discovery, and genome sequencing and selection. Such genetic resources assist in predicting the genetic value of selected candidates based on the genomic estimated breeding values (GEBV) from high-density and quality markers. Genomic selection (GS) is an approach to exploit genetic markers to develop new markers-based models to increase the genetic gain of complex traits for breeding programs. High-throughput marker technologies have changed the entire scenario of marker applications and enabled the use of GS routine work for crop improvement.

**Figure 8:**
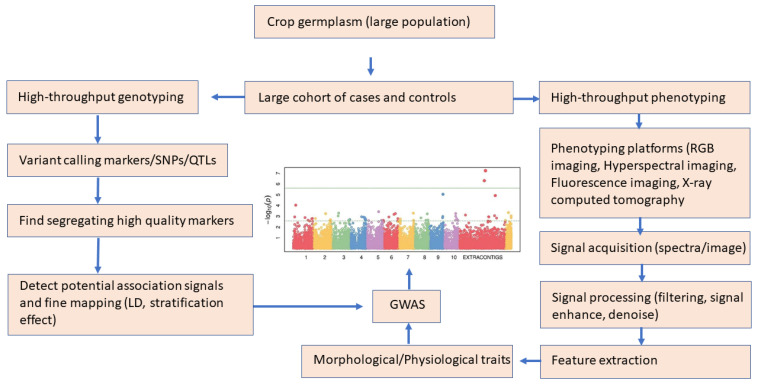
A schematic workflow of genotype and phenotype workflow for GWAS (plot adapted from [153].

Plant phenotyping through conventional methods relies on manual measurements, which are laborious, error-prone, and time-consuming. Similar to genotyping, high-throughput phenotyping (HTP) (“phenomics”) has unique advantages in facilitating accurate, automated, high-quality data collection techniques, including visible light imaging, X-ray computed tomography, visible and near-infrared spectroscopy, multispectral imaging, chlorophyll fluorescence, fluorescence imaging, and nuclear magnetic resonance (NMR) [189] (Xiao et al., 2022). These tools are generally used to obtain high-resolution images of samples from which features are extracted with image processing algorithms. Mostly machine learning algorithms are used to generate robust data processing to produce accurate and time-efficient phenotypes of plants [190]. Highly accurate genotype and phenotypic data need appropriate statistical methods to identify true associations between genetic and phenotypic variation (Figure 8). Plant phenotyping systems, imaging techniques, challenges, and their applications have been reviewed elsewhere, including imaging systems, data collection methods, and analysis techniques and problems [191,192,193]. GWAS has high efficiency and high resolution and is conducted on a genome-wide scale with statistical programs. Some of the R packages developed for association analysis are GAPIT [194], qqman [195], gwasrapidd [196], eQTpLot [197], Postgwas [198], GWASTools [199], and IntAssoPlot [200].

## 7. Data Science and Artificial Intelligence

Genomics data science is a field that needs powerful computational and statistical methods to decode the information in plant genomic DNA. Having a better understanding of genomics with these data science tools helps researchers to uncover the differences between the varieties at a DNA level and enhance crop improvement. Bioinformatics has emerged to bring in vivo experimentation and in vitro data analysis with statistical and computational tools to process the data by developing and implementing the algorithms as software tools to make predictions based on the experimental data.

Researchers are now generating more genomic data to understand genome functions and mine genetic information to explore novel insights from the vast amounts of generated genomic data. Sequencing huge numbers of individuals of a species generates terabytes of data, and processing such large amounts of data needs additional terabytes to petabytes of storage and working computational infrastructure. Researchers need special computational and software tools to mine and interpret hidden biological information through assembling the sequence data, aligning the sequence reads, and mining the variation, association studies and other genomic insights [201].

Artificial intelligence (AI) tools help researchers process vast quantities of genomic sequence data to find patterns in a genome [202]. AI typically contains hidden layers of analysis leading to biases in generating the results and may be undetected [203]. Thus, there is a need to apply human intelligence to validate the prediction/results in other dimensions.

**Figure 9:**
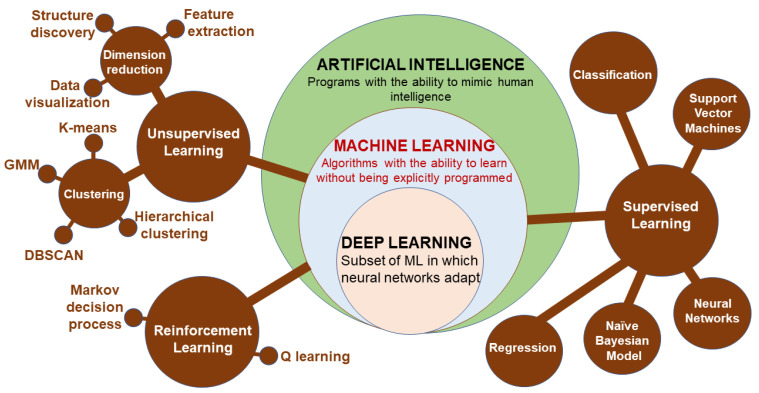
The structure of the artificial intelligence and sub-groups of machine learning methods (DBSCAN: density-based spatial clustering of applications with noise; GMM: Gaussian mixtures model).

Machine learning (ML) is a subset of artificial intelligence (AI) involving the development of algorithms that learn to perform a specific task based on given inputs. ML is implemented in either supervised learning (predicting output based on the given input features describing the object) or unsupervised learning (seeking patterns comparison and grouping the data) [204]. Supervised learning can be further grouped into two categories of algorithms: classification and regression. Similarly, unsupervised learning categories include clustering and association. Reinforcement learning is a feedback learning method in which the right action has a positive score, while a negative score is for the wrong action. The deep learning (DL) approach involves using layers of neural networks, and DL uses several such layers as artificial neural networks [204]. Convolutional neural networks are effective at image processing, while recurrent neural networks deal with sequential data and support vector machines that can capture nonlinear relations between objects. A better classification of the relationships between ML methods is depicted in Figure 9. 

ML method implementations are available in the form of a Weka (https://www.cs.waikato.ac.nz/ml/weka/) and Orange (https://orange.biolab.si/) as user-friendly graphical interfaces, scikit learn (https://scikit-learn.org/) [205], Keras [206], and PyTorch [207]. In Advances, in-neural information processing systems and are available as the TensorFlow package (https://www.tensorflow.org/overview/) [208] in Python and the Caret package in R (https://cran.r-project.org/web/packages/caret/vignettes/caret.html) [209].

ML is widely used for crop improvement; some of the case studies include plant–pathogen interactions [210], traits, and phenotyping [211], and applications include at the molecular level in plants [212]. The use of ML in plant genomics has increased in the last decade [213]; applications include the classification of genes into active and inactive genes in maize [214], identifying genome crossovers [215], identification of near-complete genetically fixed genomic regions [216], gene regulatory networks in maize [217], gene prediction with deep learning with a variety of architectures [218], diagnosis of pests and disease [219], gene prediction concerning climatic conditions [220], predicted gene expression levels from genomic sequence data [221], identifying variants based on short-read sequence alignments [222], and classifying genes as core and dispensable genes [223].

The applications of ML have been widely used in phenotyping through high-throughput, image-based plant phenotyping which uses a convolutional neural network (CNN) [224] and deep learning [225]. From a recent review [226], the most commonly used genome selection R packages based on the linear mixed model and Bayesian regression model are rrBLUP and Bayesian models rrBLUP [227], BGLR [228], lme4 [229], ASReml [230], and glmnet [231]. For the multiple trait-based genome selection, MTGS (genomic selection using multiple traits) and BMTME (Bayesian multi-trait and multi-environment) [232] packages have been developed. The more detailed approaches and categories in genome selection were discussed in an earlier study [226].

ML can improve plant breeding [204,233], with plant breeders relying on genomic selection [234] to identify the QTLs (quantitative trait loci) (genomic regions associated with traits), assess the genetic architecture of the crop, and predict traits for new genotypes. ML algorithms used for such predictions are random forests [235], support vector machines [236], and gradient tree boosting [237].

Mobile apps have been designed to collect data, record details, predict plant disease, predict weather changes, and other miscellaneous applications. The apps and the underlying algorithms interpret images captured through the devices, thus reporting the health condition of plants, soil color and other phenotypes. The availability of these apps for farmers assists in detecting disorders and suggests suitable measures to protect the crop. Some of the apps are AgSpeak (https://www.agspeak.in/), AutoML (https://www.automl.org/), aWhere (https://www.climateshot.earth/awhere), Farm at Hand (https://www.farmathand.com/), Plantix (https://plantix.net/en/), Tumaini (https://ciat.cgiar.org/phenomics-platform/tumaini/), and Xarvio (https://www.xarvio.com/global/en.html).

## 8. Conclusions/Future Aspects

The goal of improving sequencing technology has been to generate genetic information in a faster, cheaper, and more accurate way. The more portable sequencing platforms (such as the Minion from Oxford) require less power, reagents, maintenance, and storage and have an easy processing format. It is also equally important to have advanced and compatible bioinformatics tools to analyze the big data generated from the agriculture sector.

First- and second-generation sequencing technologies generated short-sequence reads resulting in highly fragmented reference genome assemblies (unless coupled with long-range systems of mapping, such as BAC-end sequencing) but were used to generate the first reference genomes for plants. Such low-quality assemblies (compared to third-generation assemblies) have many gaps and do not represent the actual genome structure. On the other hand, combined second- and third-generation sequence data have contributed to generating full chromosome-level (CL) to T2T-level reference sequences. Only in a few plant genomes have high-quality, gapless chromosome levels to T2T quality assemblies been generated; therefore, further improvements are necessary to generate high-quality standards.

T2T-level genome assemblies will provide insights into the genetic diversity, identification of domestication events, and the investigation of the evolutionary history of plant species.

The sequencing of multiple accessions of a plant species is expected to allow the assembly of a pan-genome which represents the collection of core and dispensable genes present in a species [153]. In addition to pan-genome studies, several intensive genome and transcriptome projects have been initiated (10,000 plant genomes and 1000 plant transcriptomes) [238]. Additionally, the Earth BioGenome Project (EBP) is planning to sequence and catalogue the genome of all eukaryotes on Earth.

The recent advances and developments in bioinformatics applications for plant genomes provide huge potential for plant genome research. As sequencing technology has become much more affordable and portable to handle, the importance of bioinformatics tools increases to analyze and manage the data. More plant species genome databases are being established with a variety of analysis methods. Phylogenomics and GWAS now generate more accurate results with the tools developed with newer algorithms. Moreover, high-throughput phenotyping needs to provide results with a high resolution to meet the density of genotype information.

The genetic information in the form of sequence data or optical maps needs to be as error-free as possible, selecting the appropriate informatics tools for *de novo* assembly, scaffolding, annotation, and downstream analysis. This is key for gold- or platinum-standard genome assemblies.

With the rate of the growing world population, there is a constant increase in demand for food, and AI will play a vital role in meeting these demands, coupled with computational power through robotics, smartphone apps, and image processing algorithms. AI provides automation in agriculture. Technology is being developed in agriculture for automated methods, crop improvement, and crop protection. With computational advances, including AI, ML, and DL, the future GAB, including marker-assisted selection (MAS), MABC, marker-assisted recurrent selection (MARS) [239], haplotype-based breeding, speed breeding (SB) [240,241], and genomic selection (GS), are expected to play a key role in breeding more smart crop cultivars with higher production and nutritional value in both a cost- and time-saving manner.

In the past two decades, the parallel advances in sequencing technology and bioinformatics tools have enabled plant researchers to generate genomics resources for economically important plants, which is critical for crop improvement and to develop a greater scientific understanding of the gene underlying critical traits for future agriculture. 

## Figures and Tables

**Table 2:** Recent developments and availability of plant databases.

Database Name	Description	Website	Ref
PlantcircBase	Plant circular RNAs	http://ibi.zju.edu.cn/plantcircbase/	[59]
Fine-Root Ecology Database	Fine root trait database	http://roots.ornl.gov	[60]
ATTED-II	Coexpression database	http://atted.jp	[61]
Planteome	Plant reference and species-specific ontologies for plants	http://www.planteome.org	[62]
PLADIAS	Plant diversity analysis and synthesis	www.pladias.cz	[63]
TRY plant trait database	Plant trait data	https://www.try-db.org	[64]
PmiREN	Small non-coding RNA molecules database	http://www.pmiren.com/	[65]
Plant DNA C-values	The catalogue of C-value data for land plants and algae	https://cvalues.science.kew.org/	[53]
PlantPepDB	Phyto-peptides for various therapeutic purposes	http://www.nipgr.ac.in/PlantPepDB/	[66]
MtSSPdb	*Medicago truncatula* Small Secreted Peptide Database	https://mtsspdb.noble.org/	[67]
GRooT	A collection of root traits in responses to environmental conditions	https://groot-database.github.io/GRooT/	[68]
MPDB	Medicinal plant database	https://www.medicinalplantbd.com/	[69]
GreenPhylDB	Exploration of gene families and homologous relationships among plant genomes	https://www.greenphyl.org	[70]
PlantscRNAdb	Plant single-cell RNA analysis	http://ibi.zju.edu.cn/plantscrnadb/)	[71]
TarDB	Plant miRNA target sequences	http://www.biosequencing.cn/TarDB	[72]
*Xylella* spp.	Host plant species	https://www.efsa.europa.eu/en/microstrategy/xylella	[73]
PlantGSAD	Gene set annotation plant species	http://systemsbiology.cau.edu.cn/PlantGSEAv2/	[74]
CpGDB	Plant chloroplast database	http://www.gndu.ac.in/CpGDB	[75]
DBPR	Plant protein, DNA, RNA, Pathway, and Expression Database	https://www.habdsk.org/dbpr.php	[76]
PtncRNAdb	tRNA-derived non-coding RNAs database	https://nipgr.ac.in/PtncRNAdb	[77]

**Table 3:** The pan-genome assemblies.

Approach	Species	Domestication Status	Ploidy	Number of Accessions	Reference
*de novo*	*Brassica rapa*	Crop	Diploid	3	[159]
*de novo*	*G. soya* (soybean)	Wild	Tetraploid	7	[160]
*de novo*	*O. sativa*	Crop	Diploid	3	[161]
*de novo* transcriptome	*Zea mays* (maize)	Crop	Diploid	503	[162]
*de novo* metagenome assembly	*O. sativa* (indica/japonica)	Crop	Diploid	1483	[163]
Iterative assembly	*B. oleracea*	Crop	Diploid	10	[164]
Read mapping	Populus (poplar)	Wild	Diploid	7	[165]
*de novo*	*B. distachyan*	Wild	Diploid	54	[166]
*de novo*	*Medicago truncatula*	Wild	Diploid	15	[167]
Iterative assembly	*Triticum aestivum* (bread wheat)	Crop	Hexaploid	19	[115]
Iterative assembly	*B. napus*	Crop	Tetraploid	53	[168]
Iterative assembly	*Capsicum* (pepper)	Crop	Diploid	383	[169]
Iterative assembly	*O. sativa*/*O. rufipogon*	Crop	Diploid	67	[170]
Map-to-pan	*O. sativa* (rice)	Crop	Diploid	3010	[78]
*de novo*	*Sesamum indicum* (sesame)		Diploid	5	[171]
Iterative assembly	*Helianthus annuus* (sunflower)	Crop	Diploid	493	[172]
Iterative assembly	*Solanum lycopersicum* (tomato)	Crop	Diploid	725	[116]
*de novo*	*B. napus* (oilseed rape)	Crop	Tetraploid	9	[173]
*de novo*	*Juglans* (walnut)	Wild	Diploid	6	[174]
*de novo*, graph	*G. max* (soybean)	Crop	Diploid	29	[175]
PHG	Sorghum		Diploid	398	[176]
Iterative assembly	*B. napus*	Crop	Tetraploid	50	[177]
Iterative assembly	Pigeon pea (*Cajanus cajan)*		Diploid	89	[113]
*de novo*	Pecan (*Carya illinoinensis)*	Tree	Diploid	4	[178]
*de novo*	*White lupin*	Crop	Diploid	39	[155]
Iterative assembly	Sorghum	Crop	Diploid	354	[158]
Iterative assembly	*Brassica napus*, *rapa*, *oleracea*	Crop	Diploid, diploid, amphidiploid	87, 77 and 79	[179]
Iterative assembly	*Chickpea*	Crop	Diploid	3366	[180]
*de novo*	Sorghum	Crop/Wild relatives	Diploid	16	[181]
Iterative assembly	Eggplant (*Solanum melongena* L.)		Diploid	23	[182]
Iterative assembly	Banana (*Musa* and *Ensete*)		Triploid	15	[154]
*de novo*	Tomato (*Solanum lycopersicum*)	Crop	Diploid	838	[183]
*de novo*	Potato (*Solanum tuberosum* L.)	Crop	Diploid	44	[83]
Iterative assembly	Lupin	Crop	Diploid	55	[184]

## Data Availability

Not applicable.

## Supplementary Materials

The following supporting information can be downloaded at: https://www.mdpi.com/article/10.3390/life13081668/s1, Table S1: The available plant genome assemblies (at the level of scaffold, chromosome and t2t standard quality).
